# Skeletal vascular perfusion is altered in chronic kidney disease

**DOI:** 10.1016/j.bonr.2018.05.001

**Published:** 2018-05-04

**Authors:** Mohammad W. Aref, Elizabeth A. Swallow, Neal X. Chen, Sharon M. Moe, Matthew R. Allen

**Affiliations:** a Department of Anatomy and Cell Biology, Indiana University School of Medicine, Indianapolis, IN, United States; b Department of Medicine, – Division of Nephrology, Indiana University School of Medicine, Indianapolis, IN, United States; c Department of Biomedical Engineering, Indiana University Purdue University of Indianapolis, Indianapolis, IN, United States; d Roudebush Veterans Administration Medical Center, Indianapolis, IN, United States

**Keywords:** Cortical bone perfusion, Bone marrow perfusion, Fluorescent microspheres, CKD-mineral bone disease

## Abstract

Patients with chronic kidney disease (CKD) are at an alarming risk of cardiovascular disease and fracture-associated mortality. CKD has been shown to have negative effects on vascular reactivity and organ perfusion. Although alterations in bone blood flow are linked to dysregulation of bone remodeling and mass in multiple conditions, changes to skeletal perfusion in the setting of CKD have not been explored. The goal of this study was to establish the effect of CKD on skeletal perfusion in a rat model of CKD. In two experiments with endpoints at 30 and 35 weeks of age, respectively, normal (NL) and Cy/+ (CKD) animals (*n* = 6/group) underwent *in vivo* intra-cardiac fluorescent microsphere injection to assess bone tissue perfusion. These two separate time points aimed to describe skeletal perfusion at 30 and 35 weeks based on previous studies demonstrating significant progression of hyperparthyroid bone disease during this timeframe. CKD animals had blood urea nitrogen (BUN) levels significantly higher than NL at both 30 and 35 weeks. At 30 weeks, perfusion was significantly higher in the femoral cortex (+259%, *p* < 0.05) but not in the tibial cortex (+140%, *p* = 0.11) of CKD animals relative to NL littermates. Isolated tibial marrow perfusion at 30 weeks showed a trend toward being higher (+183%, *p* = 0.08) in CKD. At 35 weeks, perfusion was significantly higher in both the femoral cortex (+173%, *p* < 0.05) and the tibial cortex (+241%, *p* < 0.05) in CKD animals when compared to their normal littermates. Isolated tibial marrow perfusion (−57%, *p* <0.05) and vertebral body perfusion (−71%, *p* <0.05) were lower in CKD animals. The current study demonstrates two novel findings regarding bone perfusion in an animal model of high turnover CKD. First, cortical bone perfusion in CKD animals is higher than in normal animals. Second, alterations in bone marrow perfision differed among the stages of CKD and were distinct from perfusion to the cortical bone. Determining whether these changes in bone perfusion are drivers, propagators, or consequences of skeletal deterioration in CKD will necessitate further work.

## 1 Introduction

Patients with chronic kidney disease (CKD) have accelerated bone loss, vascular calcification and abnormal biochemistries. Together, these factors contribute to patients being at an alarming risk of cardiovascular disease and fracture-associated mortality (Demer and Tintut, 2010). In CKD patients, the risk of cardiovascular disease is increased 3 to 100-fold (Kundhal and Lok, 2005) and the risk of fracture 4 to 14-fold (Alem et al., 2000) compared to the normal population. These risks rise progressively as kidney function deteriorates. More striking, cardiovascular disease accounts for nearly 60% of deaths in those with CKD (compared to 28% in the normal population); similarly over 60% of CKD patients that sustain a hip fracture die within a year (compared to 20% in the normal population) (Coco and Rush, 2000). These striking statistics emphasize the critical need to better understand the underlying mechanism driving altered cardiovascular and skeletal homeostasis, as well as any potential connection between the two.

Bone is a highly vascularized tissue and bone perfusion plays a crucial role in bone growth (Fleming et al., 2001), fracture repair (Tomlinson and Silva, 2014; Maes et al., 2010; Grundnes and Reikerås, 2009), and bone homeostasis (Carulli et al., 2013; McCarthy, 2006). Disturbances to bone blood flow have been shown to have associated effects on bone health and function (Carulli et al., 2013; Prisby et al., 2008; Colleran et al., 2000; Stabley et al., 2015; Stabley et al., 2013). Conditions that alter bone remodeling (diabetes, disuse, aging, estrogen withdrawal, anabolic drug treatment) have all been associated with changes in bone blood flow (Prisby et al., 2008; Colleran et al., 2000; Prisby et al., 2012; Prisby et al., 2007; Kwon et al., 2010; Bergula et al., 1999; Prisby et al., 2013; Prisby and Guignandon, 2011). Moreover, disturbances to bone vasculature, due to any of a number of causes, result in alterations in tissue perfusion (Schipani et al., 2009) and often bone loss (Arnett, 2010). CKD-induced elevations in uremic toxins have long been associated with vascular dysfunction of multiple arterial beds through endothelium-dependent, endothelium-independent and/or vascular remodeling mechanisms (Geenen et al., 2016; Palmer et al., 2011; Dhaun, 2006; Costa-Hong et al., 2009). In the setting of CKD, decreased cardiac output (Bleeker et al., 2006), vascular calcification (Moe and Chen, 2008), and endothelial dysfunction (Malyszko, 2010; Le Brocq et al., 2008; Vettoretti et al., 2006) could all contribute to altered end-organ perfusion. Surprisingly data describing alterations in skeletal vascular perfusion in the setting of CKD are lacking.

The goal of the present study was to test the hypothesis that skeletal perfusion is altered in the setting of CKD. To accomplish this goal, we utilized fluorescent microspheres, which lodge in tissue capillaries in direct proportion to the fraction of cardiac output perfusing the tissue. This technique has been shown to allow measurement of organ perfusion as effectively as radioactive microspheres (Glenny et al., 1993), the experimental gold standard (McCarthy, 2006), and has recently been applied to study skeletal perfusion in rats (Aref et al., 2017).

## 2 Methods

### 2.1 Animals

Male Cy/+ rats, Han:SPRD rats (*n* = 12) with autosomal dominant polycystic kidney disease (Moe et al., 2009a), and their unaffected (normal) littermates (*n* = 12) were used for this study. Male heterozygous rats (Cy/+) develop characteristics of CKD around 10 weeks of age that progress to terminal uremia by about 40 weeks. Our laboratory has demonstrated that this animal model recapitulates all three manifestations of CKD-Mineral and Bone Disorder (CKD-MBD) - biochemical abnormalities, extraskeletal calcification, and abnormal bone (Colleran et al., 2000; Prisby et al., 2007)(Moe et al., 2009a). There are many other animal models of the systemic repercussions of kidney disease, but unlike the Cy/+ model, most animal models of CKD are either acute injury or developmental/growth alterations and do not model the effect of the progressive nature of CKD on mineral metabolism. The model utilized in the current study (the Cy/+ rat) avoids this drawback. All animals were fed a casein diet (Purina AIN-76A, Purina Animal Nutrition, Shreevport, LA, USA); 0.53% Ca and 0.56% P from 24 weeks on during the experiment, which has been shown to produce a more consistent kidney disease in this model (Moe et al., 2009a). Blood was collected ~24 h prior to the end of the study for measurement of plasma biochemistries. All procedures were reviewed and approved by the Indiana University School of Medicine Institutional Animal Care and Use Committee prior to study initiation.

### 2.2 Experiments

CKD animals and their normal littermates were used in two separate studies, designed to assess alterations in two distinct time points along the progression of disease in the Cy/+ model:

#### 2.2.1 Experiment 1–30 week time point (~25% normal kidney function)

Normal (NL) and Cy/+ (CKD) animals (*n* = 6/group) were assessed for serum biochemistries ~ one day before undergoing *in vivo* microsphere injection to assess bone tissue perfusion.

#### 2.2.2 Experiment 2–35 week time point (~15% normal kidney function)

Normal (NL) and Cy/+ (CKD) animals (*n* = 6/group) were assessed for serum biochemistries ~ one day before undergoing *in vivo* microsphere injection to assess bone tissue perfusion.

These two separate experiments aimed to describe skeletal perfusion at 30 and 35 weeks were designed based on previous work demonstrating significant progression of skeletal disease in this timeframe (Newman et al., 2014; Moe et al., 2014). While elevations in blood urea nitrogen (BUN) are noted by 25 weeks, progressive hyperphospatemia, hyperparathyroidism, and skeletal abnormalities become evident by 30 weeks. Between 30 and 35 weeks there is marked progression of all of the end organ manifestations of CKD-MBD, including left ventricular hypertrophy, cardiac and vascular calcification, and severe high turnover bone disease evident by severe cortical porosity, high turnover and compromised mechanical properties (Newman et al., 2014; Moe et al., 2014; Hsueh et al., 2014; Moe et al., 2009b).

### 2.3 Bone perfusion measurement

Microsphere injection was performed as previously described (Aref et al., 2017). Briefly, under isoflurane anesthesia, polystyrene red fluorescent (580/605), 15 μm microspheres (FluoSpheres, ThermoFisher) were injected into the apex of the beating left ventricle after opening the chest cavity. The spheres were allowed to circulate for 60 s before the animal was euthanized by cardiac dissection. A total of 5.0 × 10^6^ spheres/kg were injected, a number sufficient to assess perfusion in skeletal tissue (Aref et al., 2017).

Tibiae, femora, humeri, vertebrae (L4 body), kidneys and testes were collected and weighed. Testes were used as a positive control for assessing adequacy of microsphere delivery within each animal. Microsphere mixing and injection was considered adequate for an animal when right and left testicle perfusions were within 25% of each other. On the basis of this criterion, no animals were excluded from the study. Femur samples were divided into proximal, middle (diaphysis), and distal segments as previously described (Colleran et al., 2000), and weighed separately. Right femoral diaphysis marrow was left intact in bone while left femoral diaphysis marrow was thoroughly flushed and femoral cortex was weighed. Marrow was extracted from the tibial diaphysis by centrifugation; both marrow and tibial cortex were weighed. Marrow was left intact in the remainder of all specimens.

Bone samples were placed in individual amber vials with 15 mL of Cal-Ex Decalcifier solution. After 7 days, decalcified bone samples were placed in 10% ethanolic postassium hydroxide (KOH) for degradation. Soft tissue samples (kidney and testes) were placed in KOH directly. After 24 h of degradation, samples were vortexed to complete the degradation process and then filtered through polyamide mesh filters (5 μm pore size). 1 mL of Cellosolve acetate (2-ethoxyethyl acetate, 98%, Sigma) was added to each of the filtered samples to dissolve the microspheres and expose the fluorescence. The 24 h KOH degradation step differed from the original protocol (Aref et al., 2017), where samples were degraded in KOH for 48 h. This slight alteration was made based on developmental work in our lab showing 24 h was sufficient for degradation with longer durations causing progressive decline in fluorescence.

All fluorescence measurements were made using the SpectraMax i3x microplate reader (Molecular Devices, CA). Three 100 μL aliquots from each sample were placed in a 96-well V-bottom polypropylene microplate for fluorescence quantification. The readings from the three aliquots were averaged to produce a single fluorescence measurement per sample. Red fluorescence was measured using an excitation of 580 nm and an emission of 620 nm. Standard curves of serial dilutions with known amounts of microspheres were generated on the day of analysis. Fluorescent measurements of samples found to be outside the standard curve (kidneys) were serially diluted and measured in order to detect any potential quenching effects. All data is presented as tissue fluorescence density (TFD) with units of Arbitrary Units per gram of tissue (AU/g) and scaled by 10^6^.

### 2.4 Biochemistries

Blood plasma was analyzed for blood urea nitrogen (BUN) and calcium using colorimetric assays (BioAssy System, DIUR-100). Intact PTH was determined by ELISA (Immutopics, REF-60-2500).

### 2.5 Statistical analysis

All analyses were performed using GraphPad Prism software. Student's *t*-tests were used to compare CKD and NL groups within each experiment. Pearson product correlations were used to assess relationships between BUN, PTH and tissue perfusion. *A priori* α-levels were set at 0.05 to determine statistical significance.

## 3 Results

### 3.1 Experiment 1: 30 week data

**Fig. 1:**
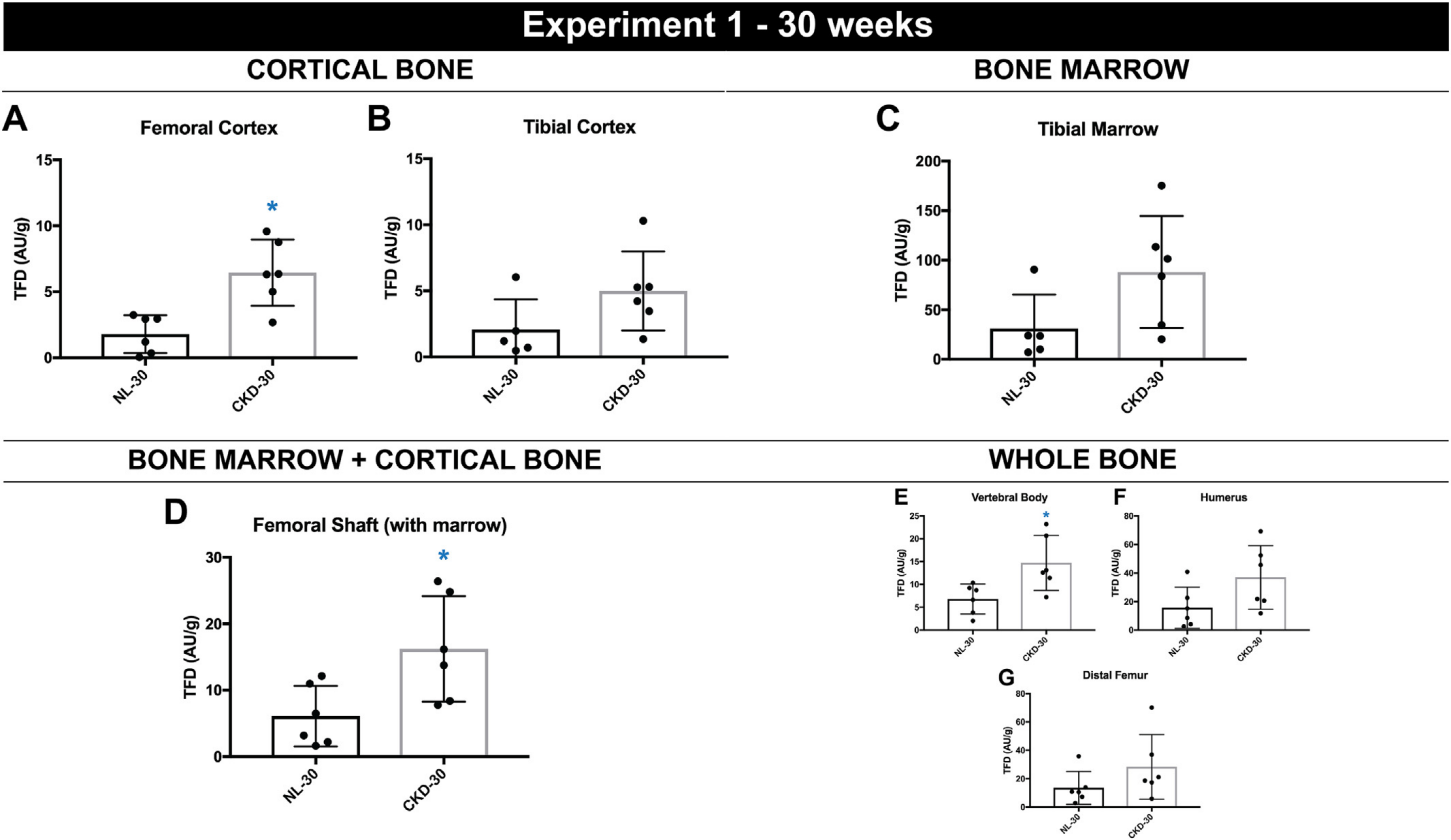
30 week time point bone perfusion data (*n* = 6/group). Tissue fluorescence density (TFD) of (A) femoral cortical bone (*p* < 0.05) (B) tibial cortical bone (*p* = 0.11) (C) tibial bone marrow (*p* = 0.08) (D) femoral diaphysis including marrow (*p* < 0.05) (E) L4 vertebral body (*p* < 0.05) (F) humerus (*p* = 0.08) and (G) distal femur (*p* = 0.18). Dots represent data points, and error bars represent standard deviation.

There was no significant difference in body or bone mass between the two groups of animals (Supplemental Tables 1 and 2). Kidney mass was significantly higher in CKD due to cystic disease compared to age-matched normal littermates (NL) (Supplemental Tables 2). Plasma BUN, but not PTH, was significantly higher in CKD compared to age-matched normal littermates (NL), the former being consistent with reduced kidney function (Table 1). TFD was significantly higher in the femoral cortex (+259%, *p* < 0.05) (Fig. 1A) but not the tibial cortex (+140%, *p* = 0.11) (Fig. 1B) of CKD animals relative to NL. Isolated tibial marrow perfusion showed a trend toward being higher (+183%, *p* = 0.08) in CKD compared to NL (Fig. 1C). Vertebral body TFD was significantly higher in CKD animals (+116%, *p* < 0.05) while neither distal femur (+109%, *p* = 0.18) or humerus (+136%, *p* = 0.08), significantly differed between groups (Fig. 1E–G). These three bone sites all had intact marrow. Kidney perfusion was not significantly different in CKD animals when compared to their normal littermates at 30 weeks (*p* = 0.06) (Supplemental Table 3). There were no scientifically significant correlations between PTH and TFD for either NL or CKD animals (data not shown).

**Table 1:** Serum biochemistries.

	NL	CKD
Experiment 1–30 weeks		
BUN, (mg/dL)	19.1 ± 1.7	39.7 ± 6.0⁎
PTH, (pg/mL)	376 ± 298	420 ± 378

Experiment 2–35 weeks		
BUN, (mg/dL)	17.8 ± 1.9	50.4 ± 8.0⁎
PTH, (pg/mL)	123 ± 49	1305 ± 237⁎

Data presented as mean and standard deviation.

⁎ *p* < 0.05.

### 3.2 Experiment 2: 35 week data

**Fig. 2:**
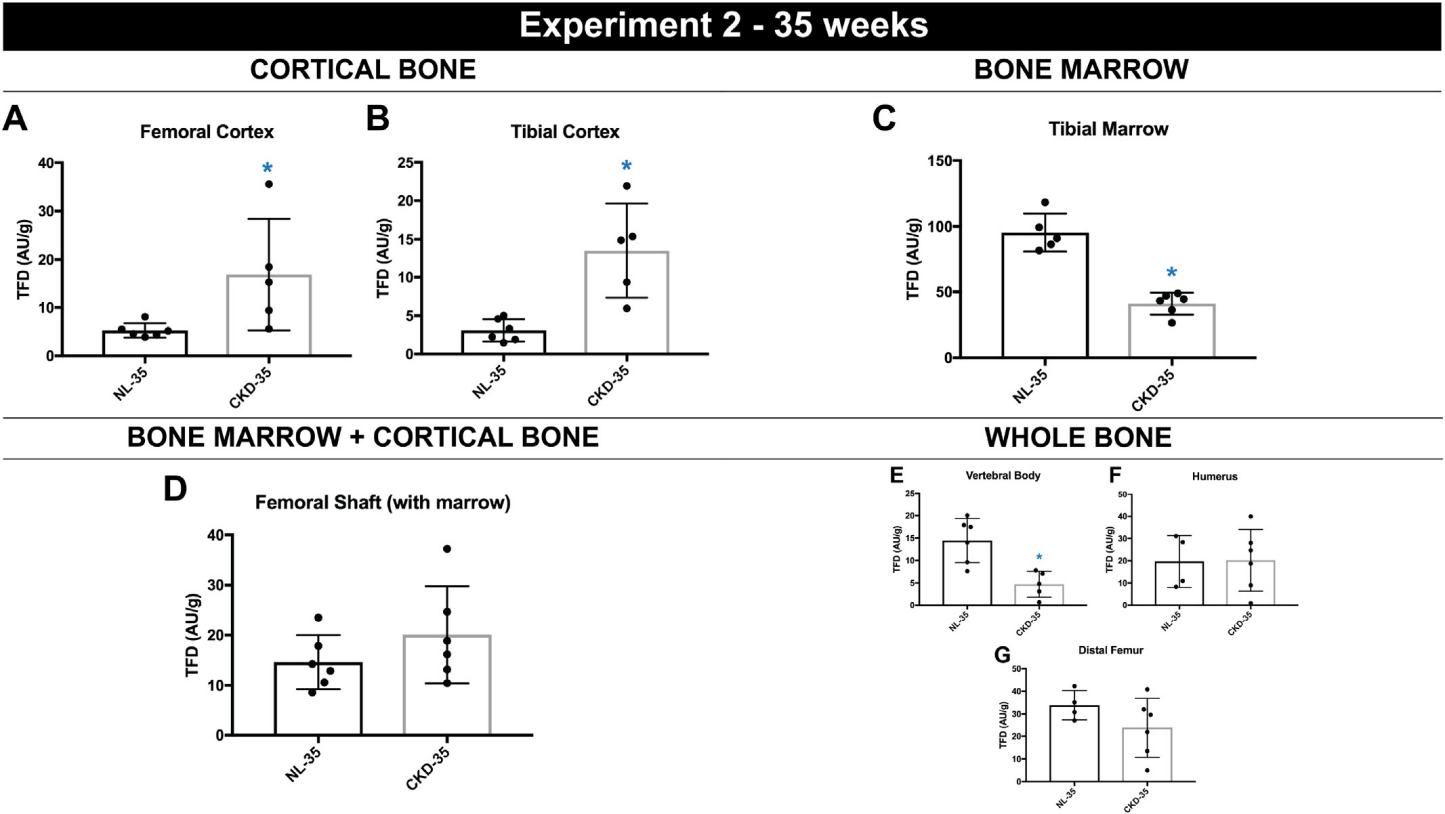
35 week time point bone perfusion data (*n* = 6/group). Tissue fluorescence density (TFD) of (A) femoral cortical bone (*p* < 0.05) (B) tibial cortical bone (*p* < 0.05) (C) tibial bone marrow (*p* < 0.05) (D) femoral diaphysis including marrow (*p* < 0.05) (E) L4 vertebral body (*p* < 0.05) (F) humerus (*p* = 0.95) and (G) distal femur (*p* = 0.17). Dots represent data points, and error bars represent standard deviation.

Animal body mass was significantly lower in CKD (−15%) compared to NL animals (Supplemental Table 1). Kidney mass was significantly higher and femoral diaphysis (with marrow) mass was significantly lower in CKD compared to age-matched normal littermates (NL) (Supplemental Table 2). Plasma BUN and PTH were both significantly higher in CKD compared to NL (Table 1). TFD in CKD animals was significantly higher in both the femoral cortex (+173%, *p* < 0.05) (Fig. 2A) and the tibial cortex (+241%, *p* < 0.05) (Fig. 2B) relative to NL. Isolated tibial marrow TFD was significantly lower (−57%, *p* < 0.05) in CKD animals when compared to age-matched normal littermates (Fig. 2C). Vertebral body perfusion (−71%, *p* < 0.05) was significantly lower in CKD animals compared to NL while neither distal femur (−27%%, *p* = 0.17) or humerus (−10%, *p* = 0.95) perfusions, both with marrow intact, were significantly different between groups (Fig. E–G). Kidney perfusion was significantly lower in CKD animals when compared to their normal littermates (*p* < 0.05) (Supplemental Table 3). There was no significant correlation between PTH and TFD for any site in the NL animals while 4 of the 6 sites assessed for TFD had significant negative relationships with PTH values (Table 2).

**Table 2:** 35 week correlations between PTH and tissue fluorescence density.

	Femoral cortex TFD	Tibial cortex TFD	Tibial marrow TFD	Distal femur TFD	L4 TFD	Humerus TFD
PTH (NL)	0.337	−0.264	−0.617	0.294	0.465	0.465
PTH (CKD)	**−0.764**	**−0.772**	0.338	**−0.888**	0.653	**−0.724**

Data presented at r values with bolded values indicating *p* < 0.05.

## 4 Discussion

Deterioration of both bone and cardiovascular properties have been well documented during the progression of CKD. Bone is a highly vascularized tissue that depends on regulated perfusion for growth, repair, and homeostasis (Marenzana and Arnett, 2013). Since CKD is known to be associated with both cardiac and vascular abnormalities, the investigation of skeletal perfusion in the setting of CKD could provide insights into the pathophysiology of abnormal bone in CKD. The current study demonstrates two findings regarding bone perfusion in an animal model of high turnover CKD. First, cortical bone perfusion is higher than it is in animals with normal kidney function. Second, changes in bone marrow perfusion are more complex than those of bone, with higher perfusion early in disease and lower levels with prolonged/late stage disease. The differential changes in bone and marrow perfusion likely account for the more modest differences between CKD and NL in bone segments containing both tissues (Fig. 1, Fig. 2). The opposite trends of cortical bone and bone marrow and the proportional amount and type of marrow in each of the tested whole bones may play a role in the unclear trend observed in whole bone at 35 weeks.

Using fluorescent microspheres to measure regional bone perfusion, we show that animals with high turnover CKD have higher cortical bone perfusion at both 30 and 35 weeks compared to normal. Despite evidence of vascular pathologies in the current model (Moe et al., 2009a; Hsueh et al., 2014; Moe et al., 2009b) and known vascular dysfunction in CKD (Gansevoort et al., 2013; Haydar et al., 2004; Ameer et al., 2015), we show that cortical bone perfusion in isolated femoral and tibia cortical bone diaphyses is nonetheless higher. We hypothesize that this elevated cortical perfusion is due to one, or a combination, of two separate mechanisms. Cortical perfusion may be increased in response to increased metabolic needs of high turnover CKD bone, necessitating endothelial cells to express vasoactive substances that increase tissue blood flow (Adair et al., 1990). Alternatively, PTH has been shown to have direct effects on the endothelial expression of vascular endothelial growth factor (Rashid et al., 2008) such that worsening secondary hyperparathyroidism could be driving increased perfusion.

Conditions that alter bone remodeling (diabetes, disuse, aging, estrogen withdrawal, anabolic drug treatment) have all been associated with changes in bone blood flow (Prisby et al., 2008; Colleran et al., 2000; Prisby et al., 2012; Prisby et al., 2007; Kwon et al., 2010; Bergula et al., 1999; Prisby et al., 2013; Prisby and Guignandon, 2011). Previous work has demonstrated that changes in perfusion can precede alterations to bone structure and function in these models. Increased perfusion occurs prior to fatigue loading-induced addition of bone mass (Matsuzaki et al., 2007). By 30 weeks in this model Cy/+ rats have significant elevations in bone remodeling on trabecular bone surfaces whereas by 35 weeks they not only have high remodeling but also significant increases in intracortical remodeling and peritrabecular fibrosis. Previous work from our group suggests the escalation of skeletal deterioration in terms of increased turnover, impaired mechanics, cortical porosity, loss of cortical mass, and increased marrow fibrosis in the Cy/+ rat model between the two time points evaluated in this study – 30 and 35 weeks (Moe et al., 2014; Allen et al., 2013). Further investigations will be needed to determine whether blood flow changes are driven by metabolic demands in CKD and whether these drive the skeletal phenotype (cortical porosity) or whether the bone and/or marrow changes alter the vascular perfusion.

In the setting of CKD, we and others have shown that sustained elevated PTH contributes to high bone remodeling which drives increases in cortical porosity and ultimately compromised bone mechanics (Newman et al., 2014; Moe et al., 2014) but its contribution in vascular perfusion in CKD is unknown. The direct role of PTH in modulating vasculature, including that of the bone, has been well-established in the literature (Prisby and Guignandon, 2011; Roche et al., 2014; Rostand and Drüeke, 1999; Wang et al., 1993). Early studies illustrated the acute effects of intravenous injection of PTH to increase tibial and femoral perfusion within 30 min after administration of intravenous PTH (Kapitola and Zák, 2003). This suggested a vasodilatory effect that was confirmed in a recent study that showed PTH enhanced endothelium-dependent vasodilation of the femoral principal nutrient artery *via* augmented nitric oxide production (Prisby et al., 2013). Both of these studies represent acute PTH, and the effects of chronic elevation of PTH as seen in CKD may be different, given the divergent effects of intermittent and continuous PTH on bone mass. Roche et al. found intermittent PTH stimulated bone formation and prevented OVX-induced reduction in bone perfusion and bone vessel density, while continuous PTH resulted in a decrease in vessel size (Roche et al., 2014). Another study showed that treatment with teriparatide resulted an increase in bone blood flow, evaluated for up to 18 months (Moore et al., 2010). Our correlation analysis of PTH and tissue perfusion resulted in an unexpected strong negative relationship between PTH and tissue perfusion across multiple bones. While these data cannot speak to cause/effect, they provide a basis for future hypotheses that can fuel studies aimed at dissecting the role of PTH levels in CKD-related skeletal perfusion changes.

Patterns of marrow perfusion (marrow having been extracted from the diaphyseal region only) in CKD animals diverged from those of cortical bone in late-stage high turnover disease. Although CKD animals show no change to marrow perfusion (trending toward higher) in the 30 week time point there was significantly lower perfusion at 35-weeks compared to NL animals. This is in contrast to cortical bone perfusion which was significantly higher in CKD animals at both of the time points. Previous work from our group has demonstrated lower levels of VEGF-A expression in bone marrow of 35-week old CKD animals compared to their normal littermates (Chen et al., 2015). These suggest there may either be a dramatic shift in marrow VEGF signaling or marrow content during the later-stage manifestation of CKD. Given the known fibrosis that occurs with the severe hyperparathyroid bone disease osteitis fibrosis cystica this may have decreased the overall non-fibrotic marrow in the 35 weeks animals. An alternative explanation is that more severe cardiac dysfunction due to heart calcification or aorta calcification may limit the ability to perfusion distal organs such as bone at late stage CKD (Moe et al., 2009b).

Our results should be interpreted in the context of various assumptions and limitations. Injection of microspheres in the left ventricle to assess perfusion is based on a set of assumptions, including: microspheres are homogeneously distributed in the left ventricle, trapped in capillaries on first passage with no shunting or dislodging, and do not themselves alter the hemodynamics upon injection. This is the same set of assumptions made in any blood flow measurement using microspheres, the current experimental gold standard for the determination of skeletal perfusion. A recovery standard was not utilized in order to ensure that sample is not lost during processing. The animals are anesthetized using isoflurane, which is known to affect organ perfusion (Bernard et al., 1991) and cardiovascular dynamics (Bernard et al., 1990). Without the use of assisted ventilation, the open-chest cardiac injection of microspheres is performed under diminishing physiologic hemodynamic, as well as hypoxic, conditions. Given that the time from anesthesia to injection is consistent in experiments at each time point, and the injected spheres are fully circulated within the 60 s between injection and euthanasia, declining kidney function is not a major factor in the differences detected by our perfusion measurements.

In conclusion, we have shown that bone perfusion is altered in an animal model of progressive high turnover chronic kidney disease. Determining whether these changes in bone perfusion are drivers, propagators, or consequences of skeletal deterioration in CKD will necessitate further work.

## Transparency document

Transparency document Image 1

## Appendix A Supplementary data

Supplementary tables Image 2
