# Supplementary material for: Skeletal vascular perfusion is altered in chronic kidney disease
Source: Bone Rep. 2018 May 4;8:215–20. doi: 10.1016/j.bonr.2018.05.001 (PMC6020396; doi:10.1016/j.bonr.2018.05.001)
Supplement: Supplementary file 2 — Supplementary tables [file mmc2.docx]

**Supplemental Table 1:** Body Masses

|  | 30 weeks | | 35 weeks | |
| --- | --- | --- | --- | --- |
|  | NL | CKD | NL | CKD |
| Body Mass (grams) | 541.3 ± 34.7 | 531.8 ± 15.1 | 590.2 ± 34.1 | 500.5 ± 68.5* |

Data presented as mean and standard deviation. *p<0.05

**Supplemental Table 2:** Tissue Masses

|  | Tissue Mass | | | |
| --- | --- | --- | --- | --- |
|  | 30 weeks | | 35 weeks | |
|  | NL | CKD | NL | CKD |
| Kidney (grams) | 1.95 ± 0.10 | 3.27 ± 0.22* | 1.79 ± 0.31 | 3.69 ± 1.07* |
| Tibial Cortex (grams) | 0.19 ± 0.01 | 0.19 ± 0.02 | 0.21 ± 0.02 | 1.79 ± 0.31 |
| Tibial Marrow (grams) | 0.029 ± 0.01 | 0.031 ± 0.01 | 0.039 ± 0.01 | 0.038 ± 0.01 |
| Femoral Cortex (grams) | 0.34 ± 0.03 | 0.32 ± 0.04 | 0.28 ± 0.04 | 0.31 ± 0.02 |
| Femoral Diaphysis (with marrow) (grams) | 0.36 ± 0.02 | 0.35 ± 0.02 | 0.36 ± 0.02 | 0.31 ± 0.03* |
| Distal Femur (grams) | 0.62 ± 0.03 | 0.63 ± 0.04 | 0.69 ± 0.04 | 0.65 ± 0.07 |
| L4 – Vertebral Body (grams) | 0.28 ± 0.04 | 0.25 ± 0.02 | 0.23 ± 0.05 | 0.23 ± 0.02 |
| Humerus (grams) | 0.72 ± 0.03 | 0.71 ± 0.04 | 0.73 ± 0.05 | 0.71 ± 0.23 |

Data presented as mean and standard deviation. *p<0.05

**Supplemental Table 3:** Kidney Perfusion

|  | 30 weeks | | 35 weeks | |
| --- | --- | --- | --- | --- |
|  | NL | CKD | NL | CKD |
| Kidney Perfusion (TFD) | 562.5 ± 424.8 | 178.2 ± 106.0 | 1039.7 ± 259.3 | 66.1 ± 32.3* |

Data presented as mean and standard deviation. *p<0.05; TFD = tissue fluorescent density (AU/grams)
